# Supplementary material for: The predicted secreted proteome of activated sludge microorganisms indicates distinct nutrient niches
Source: mSystems. 2024 Sep 10;9(10):e00301-24. doi: 10.1128/msystems.00301-24 (PMC11495043; doi:10.1128/msystems.00301-24)
Supplement: Supplemental Figures — Figures S1–S7. [file msystems.00301-24-s0004.pdf]

Supp. Fig. 1. Relationships of counts of proteins from different predicted subcellular locations per MAG with MAG sizes. Each dot represents a MAG. A) extracellular, B) outermembrane and cell wall, C) periplasmic, D) cytoplasmic membrane, E) unknown with signal peptides, and F) cytoplasmic.

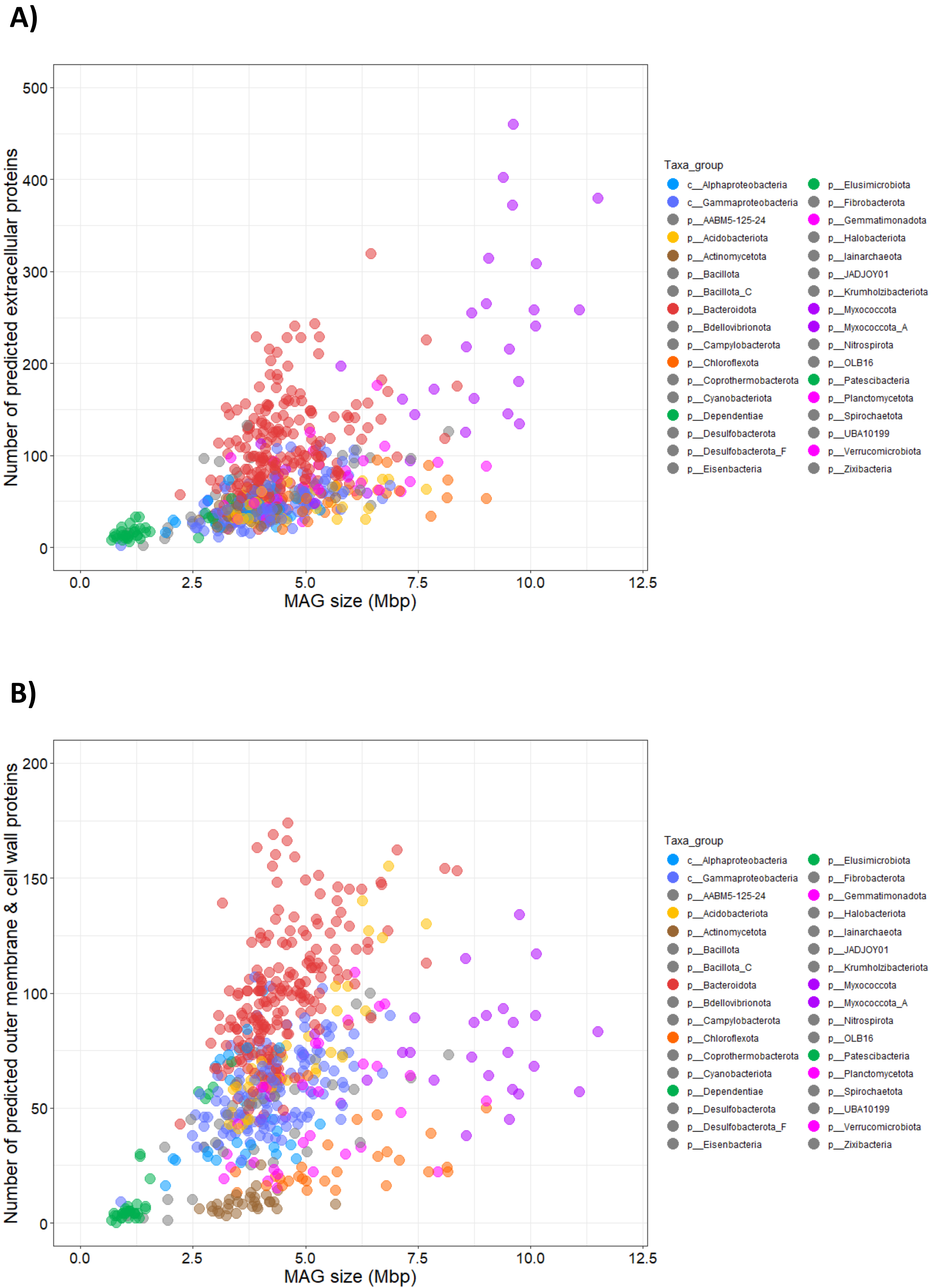
A)
Number of predicted extracellular proteins
MAG size (Mbp)
B)
Number of predicted outer membrane & cell wall proteins
MAG size (Mbp)
Taxa_group
c__Alphaproteobacteria
c__Gammaproteobacteria
p__AABM5-125-24
p__Acidobacteriota
p__Actinomycetota
p__Bacillota
p__Bacillota_C
p__Bacteroidota
p__Bdellovibrionota
p__Campylobacterota
p__Chloroflexota
p__Coprothermobacterota
p__Cyanobacteriota
p__Dependentiae
p__Desulfobacterota
p__Desulfobacterota_F
p__Eisenbacteria
p__Elusimicrobiota
p__Fibrobacterota
p__Gemmatimonadota
p__Halobacteriota
p__Iainarchaeota
p__JADJOY01
p__Krumholzibacteriota
p__Myxococcota
p__Myxococcota_A
p__Nitrospirota
p__OLB16
p__Patescibacteria
p__Planctomycetota
p__Spirochaetota
p__UBA10199
p__Verrucomicrobiota
p__Zixibacteria

C)

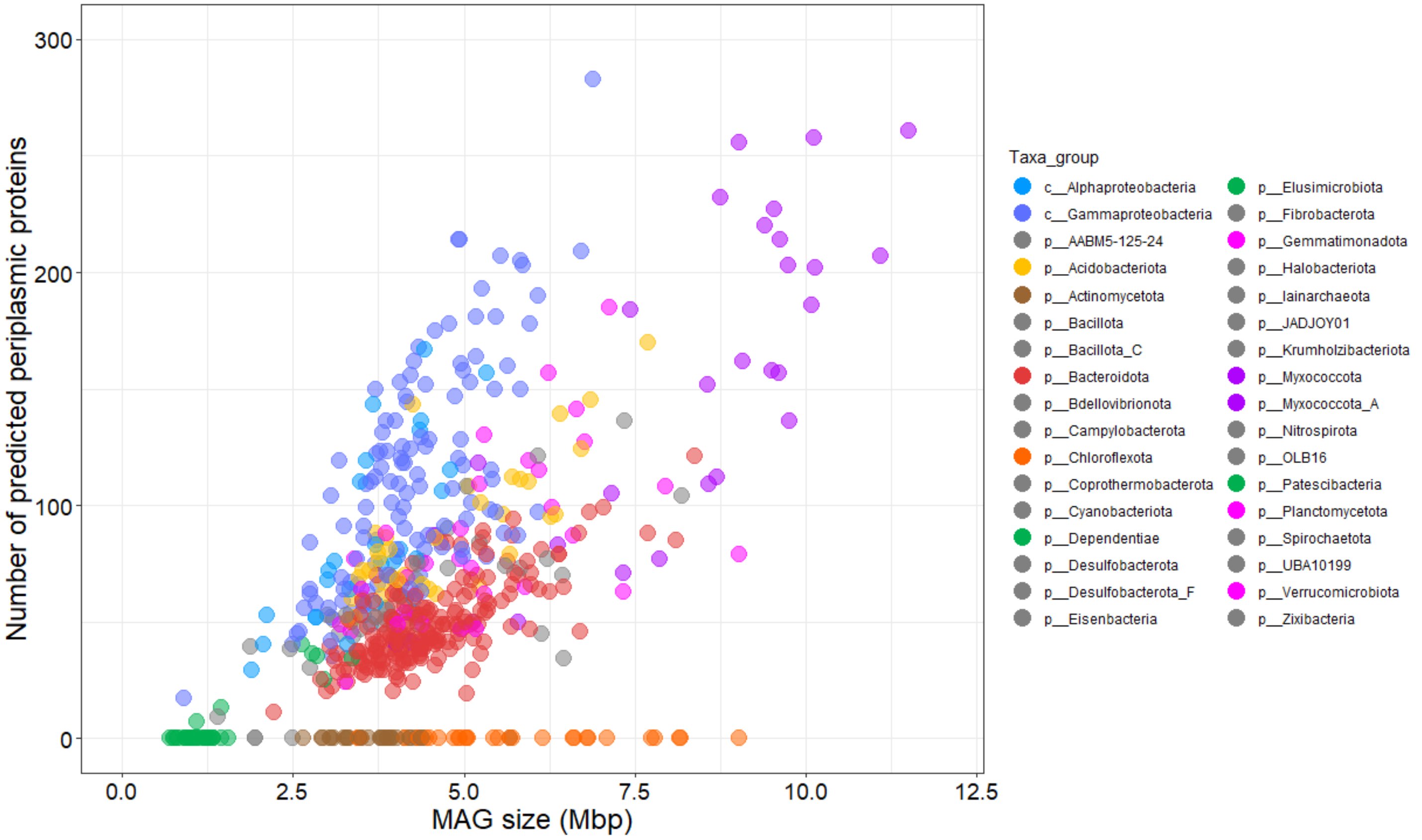

Number of predicted periplasmic proteins
MAG size (Mbp)
0
100
200
300
0.0
2.5
5.0
7.5
10.0
12.5
Taxa_group
c__Alphaproteobacteria
c__Gammaproteobacteria
p__AABM5-125-24
p__Acidobacteriota
p__Actinomycetota
p__Bacillota
p__Bacillota_C
p__Bacteroidota
p__Bdellovibrionota
p__Campylobacterota
p__Chloroflexota
p__Coprothermobacterota
p__Cyanobacteriota
p__Dependentiae
p__Desulfobacterota
p__Desulfobacterota_F
p__Eisenbacteria
p__Elusimicrobiota
p__Fibrobacterota
p__Gemmatimonadota
p__Halobacteriota
p__Iainarchaeota
p__JADJOY01
p__Krumholzibacteriota
p__Myxococcota
p__Myxococcota_A
p__Nitrospirota
p__OLB16
p__Patescibacteria
p__Planctomycetota
p__Spirochaetota
p__UBA10199
p__Verrucomicrobiota
p__Zixibacteria

D)

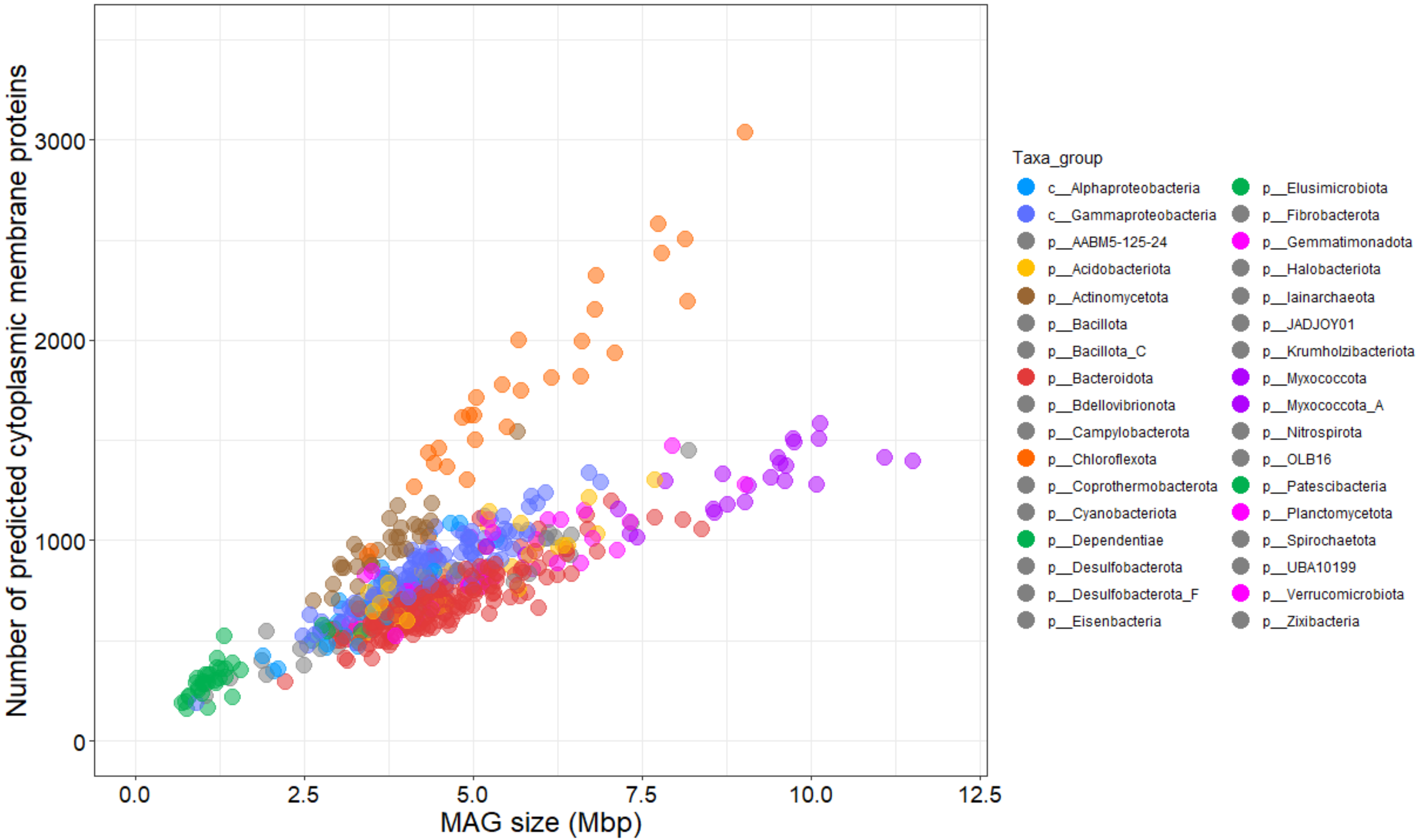

Number of predicted cytoplasmic membrane proteins
MAG size (Mbp)
0
1000
2000
3000
0.0
2.5
5.0
7.5
10.0
12.5
Taxa_group
c__Alphaproteobacteria
c__Gammaproteobacteria
p__AABM5-125-24
p__Acidobacteriota
p__Actinomycetota
p__Bacillota
p__Bacillota_C
p__Bacteroidota
p__Bdellovibrionota
p__Campylobacterota
p__Chloroflexota
p__Coprothermobacterota
p__Cyanobacteriota
p__Dependentiae
p__Desulfobacterota
p__Desulfobacterota_F
p__Eisenbacteria
p__Elusimicrobiota
p__Fibrobacterota
p__Gemmatimonadota
p__Halobacteriota
p__Iainarchaeota
p__JADJOY01
p__Krumholzibacteriota
p__Myxococcota
p__Myxococcota_A
p__Nitrospirota
p__OLB16
p__Patescibacteria
p__Planctomycetota
p__Spirochaetota
p__UBA10199
p__Verrucomicrobiota
p__Zixibacteria

E)

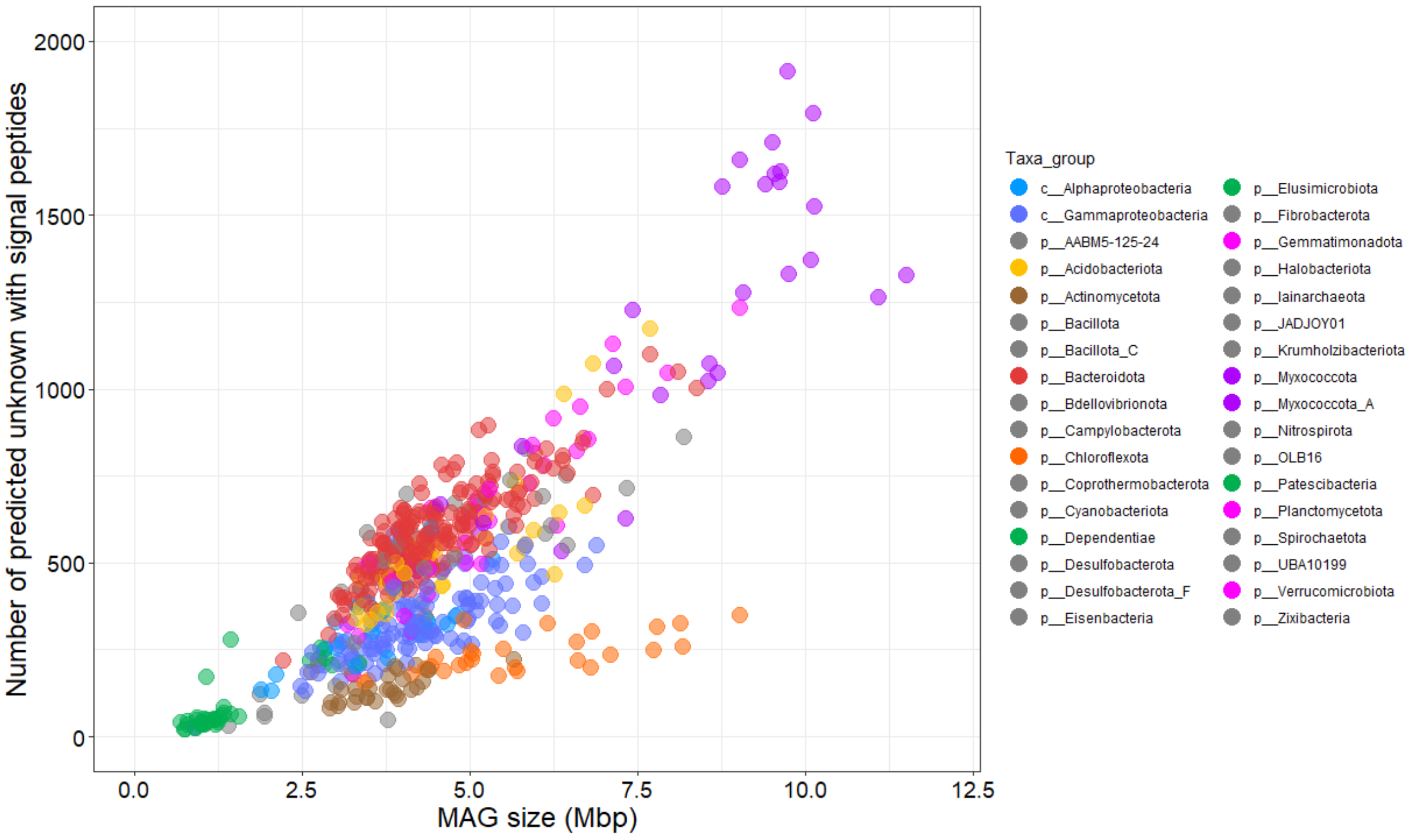

Number of predicted unknown with signal peptides
MAG size (Mbp)
0
500
1000
1500
2000
0.0
2.5
5.0
7.5
10.0
12.5
Taxa_group
c__Alphaproteobacteria
c__Gammaproteobacteria
p__AABM5-125-24
p__Acidobacteriota
p__Actinomycetota
p__Bacillota
p__Bacillota_C
p__Bacteroidota
p__Bdellovibrionota
p__Campylobacterota
p__Chloroflexota
p__Coprothermobacterota
p__Cyanobacteriota
p__Dependentiae
p__Desulfobacterota
p__Desulfobacterota_F
p__Eisenbacteria
p__Elusimicrobiota
p__Fibrobacterota
p__Gemmatimonadota
p__Halobacteriota
p__Iainarchaeota
p__JADJOY01
p__Krumholzibacteriota
p__Myxococcota
p__Myxococcota_A
p__Nitrospirota
p__OLB16
p__Patescibacteria
p__Planctomycetota
p__Spirochaetota
p__UBA10199
p__Verrucomicrobiota
p__Zixibacteria

F)

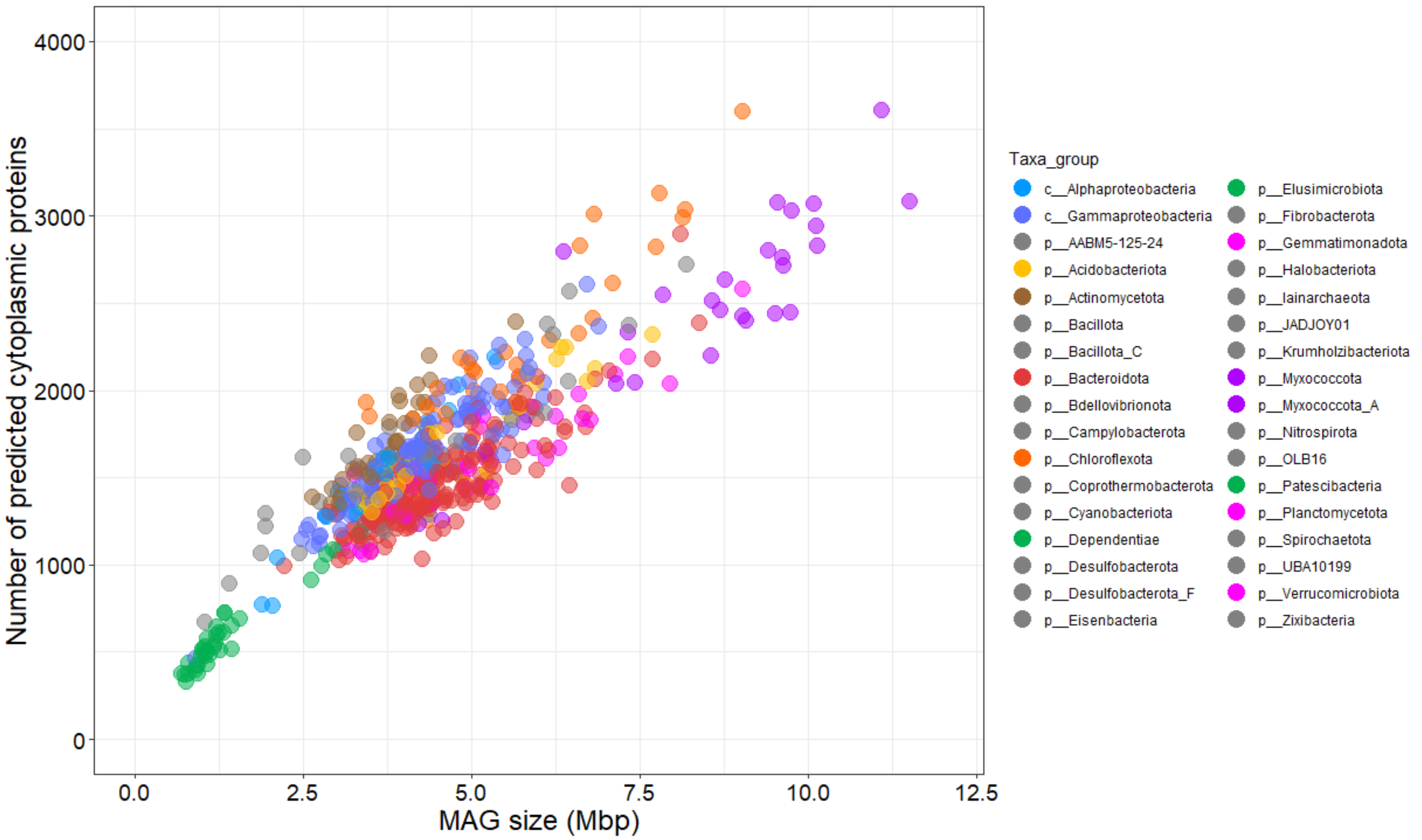

Number of predicted cytoplasmic proteins
MAG size (Mbp)
0
1000
2000
3000
4000
0.0
2.5
5.0
7.5
10.0
12.5
Taxa_group
c__Alphaproteobacteria
c__Gammaproteobacteria
p__AABM5-125-24
p__Acidobacteriota
p__Actinomycetota
p__Bacillota
p__Bacillota_C
p__Bacteroidota
p__Bdellovibrionota
p__Campylobacterota
p__Chloroflexota
p__Coprothermobacterota
p__Cyanobacteriota
p__Dependentiae
p__Desulfobacterota
p__Desulfobacterota_F
p__Eisenbacteria
p__Elusimicrobiota
p__Fibrobacterota
p__Gemmatimonadota
p__Halobacteriota
p__Iainarchaeota
p__JADJOY01
p__Krumholzibacteriota
p__Myxococcota
p__Myxococcota_A
p__Nitrospirota
p__OLB16
p__Patescibacteria
p__Planctomycetota
p__Spirochaetota
p__UBA10199
p__Verrucomicrobiota
p__Zixibacteria

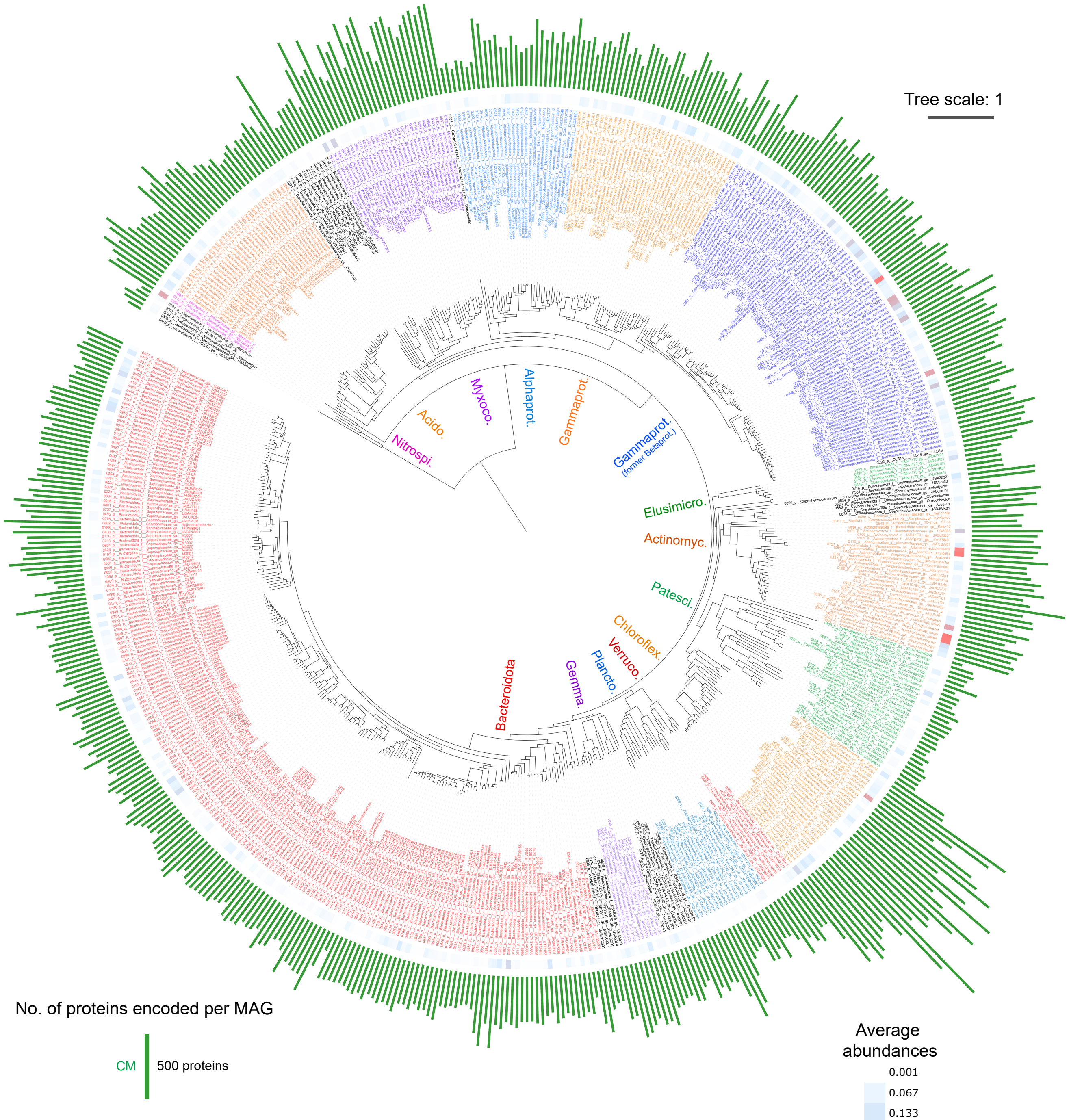

Supp. Fig. 2. Phylogenomic tree of 581 MAGs from Danish WWTPs with counts of predicted cytoplasmic memrane proteins. Green bars correspond to counts of predicted cytoplasmic membrane proteins. Most inner rign ("Abund.") with heatmap corresponds to average relative abundances of MAG-populations based on read mapping to MAGs from all metagenomes analysed (values also in Supp. Table 1, colour-scale presented in legend to bottom-right). Leaf labels include the MAG number, followed by taxonomic strings of: phyla (class for Pseudomonadota), family, genus-species, denoted by p__, c__, f__, gs__, respectively. Clades of most major phyla are indicated inside the tree with: Nitrospirota; Acidobact. (Acidobacteriota); Myxococc. (Myxococcota); Alphaprot. (Alphaproteobacteria); Gammaprot. (Gammaproteobacteria); Betaprot. (Betaproteobacteria); Elusimicro. (Elusimicrobiota); Actinomyc. (Actinomycetota); Patesci. (Patescibacteria); Chloroflex. (Chloroflexota), Verruco. (Verrucomicrobiota); Plancto. (Planctomycetota); Gemma. (Gemmatimonadota); Bacteroidota. GTDB species names are only presented if named, i.e., GTDB number codes were removed. The tree is based on a concatenated alignment of protein sequences derived from single copy marker genes obtained from CheckM analysis of MAGs. Scale bar represents 100% sequence divergence.

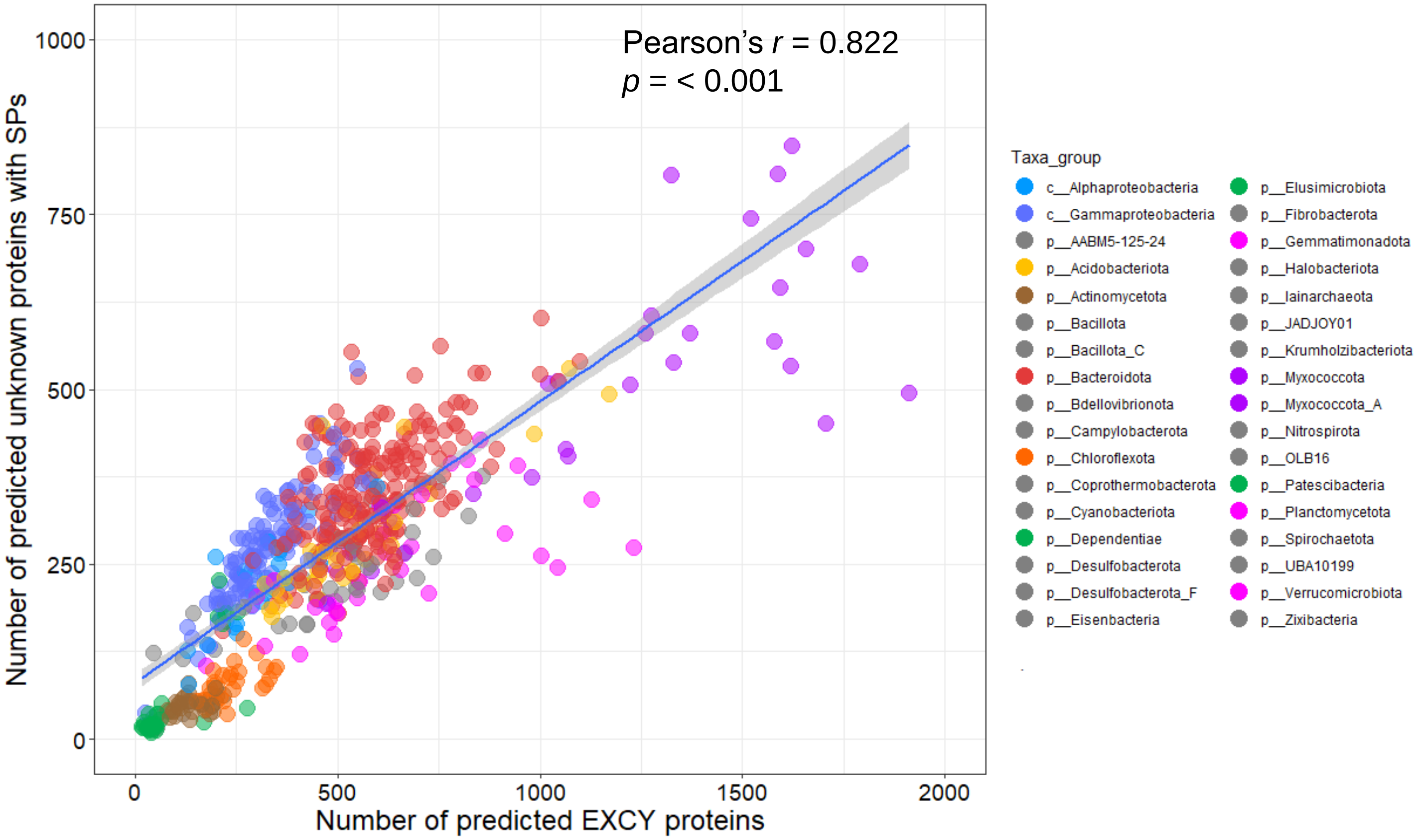

Supp. Fig. 3. Correlation of counts of predicted EXCY proteins with those with unknown locations but having signal peptides. "EXCY" = all predicted extracellular, cell wall, outermembrane and periplasmic proteins.

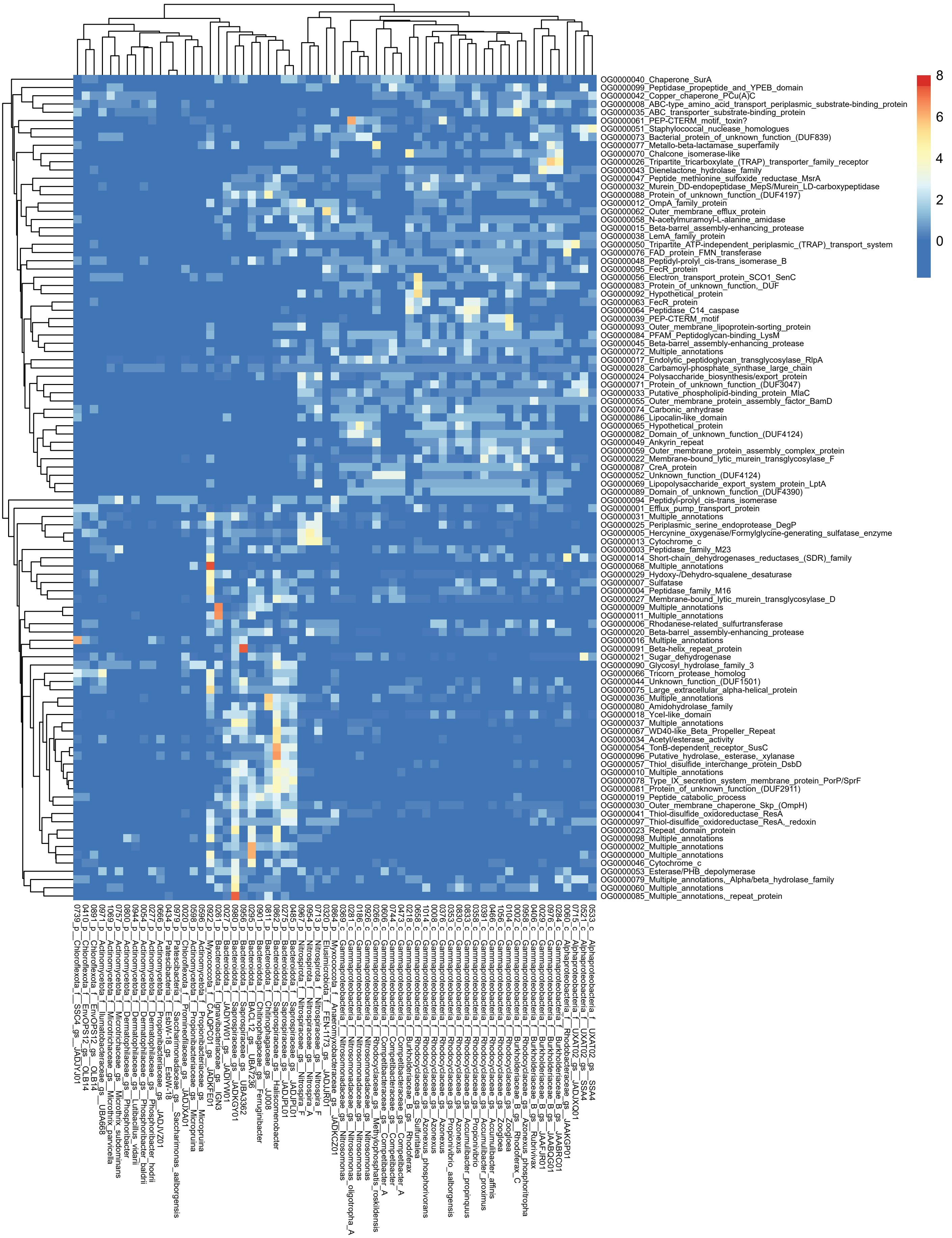

Supp. Fig. 4. Heatmap and cluster analysis of proteins from orthogroup analysis of proteins with unknown locations with signal peptides from abundant and functionally relevant taxa. Rows are centered; unit variance scaling is applied to rows. Both rows and columns are clustered using correlation distance and average linkage. The scale for the heatmap colours are indicated in the legend, where the scale maximum of 8 was used to enhance visualisation and differentiation of lower values <8. Note that some values were therefore >8, and raw values of OG counts are available in Supp. Table 3. MAG labels include the MAG number, followed by taxonomic strings of: phyla (class for Pseudomonadota), family, genus-species, denoted by p__, c__, f__, gs__, respectively.

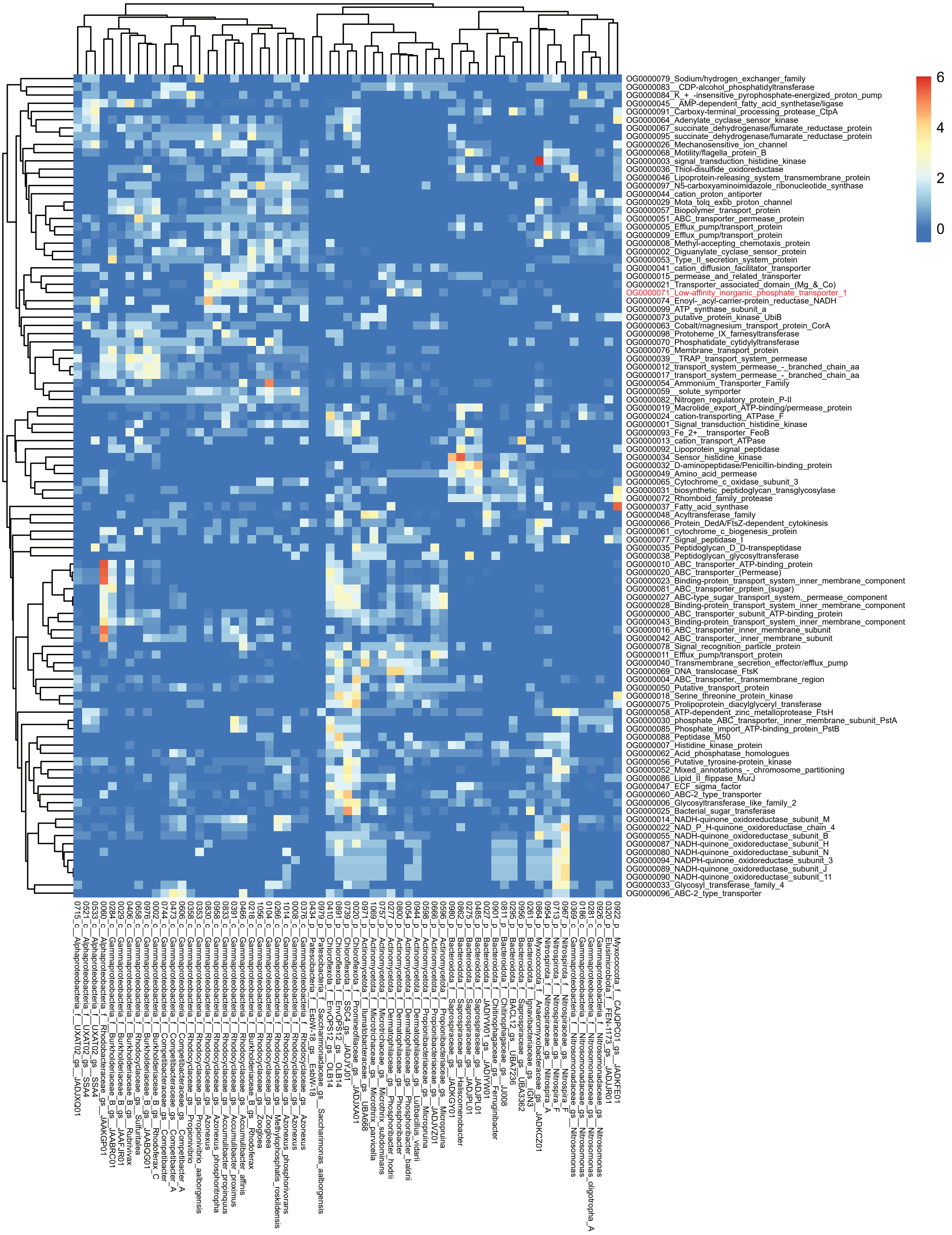

Supp. Fig. 5. Heatmap and cluster analysis of proteins from orthogroup analysis of proteins with predicted cytoplasmic membrane locations from abundant and functionally relevant taxa. Rows are centered; unit variance scaling is applied to rows. Both rows and columns are clustered using correlation distance and average linkage. The scale for the heatmap colours are indicated in the legend, where the scale maximum of 6 was used to enhance visualisation and differentiation of lower values <6. Note that some values were therefore >6, and raw values of OG counts are available in Supp. Table 4. MAG labels include the MAG number, followed by taxonomic strings of: phyla (class for Pseudomonadota), family, genus-species, denoted by p__, c__, f__, gs__, respectively.

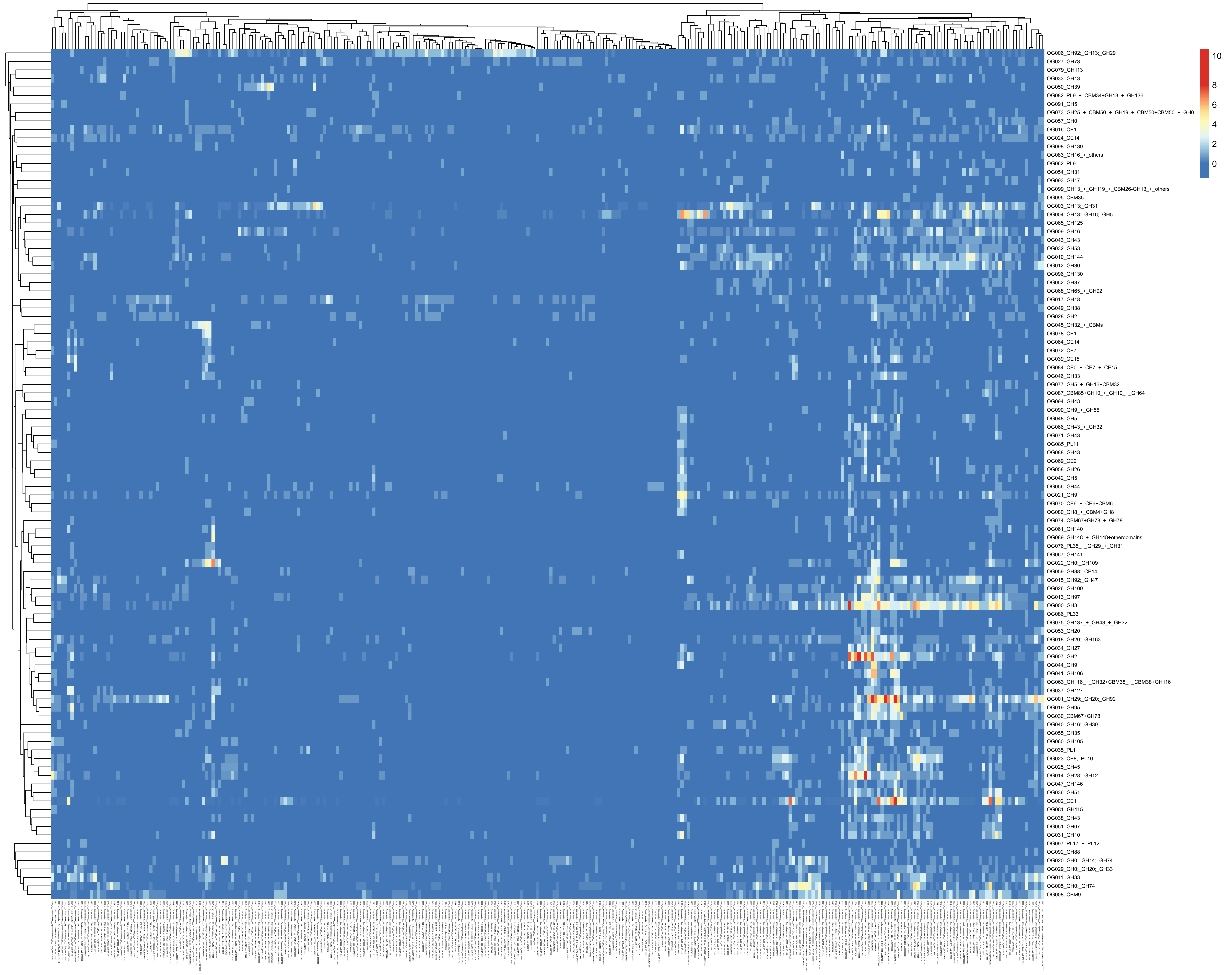

Supp. Fig. 6. Heatmap and cluster analysis of proteins from ortholog-group (OG) analysis of predicted secreted CAZymes from MAGs of phyla that contain potential key polysaccharide degraders (i.e., Bacteroidota, Acidobacteroidota, Gemmatimonadota, Verrucomicrobiota, Planctomycetota, Fibrobacterota, Chloroflexota, Cellvibrio, Krumholzibacteriota) (*N* = 303). This included proteins from all extra-cytoplasmic locations and those with Unknown locations plus signal peptides. Rows are centered; unit variance scaling is applied to rows. Both rows and columns are clustered using correlation distance and average linkage. The scale for the heatmap colours are indicated in the legend, where the scale maximum of 8 was used to enhance visualisation and differentiation of lower values <8. Note that some values were therefore >8, and raw values of OG counts are available in Supp. Table 10. The protein sequences and CAZyme annotations of the OGs are provided in Supplement Data file 3. MAG labels include the MAG number, followed by taxonomic strings of: phyla (class for Pseudomonadota), family, genus-species, denoted by p__, c__, f__, gs__, respectively.

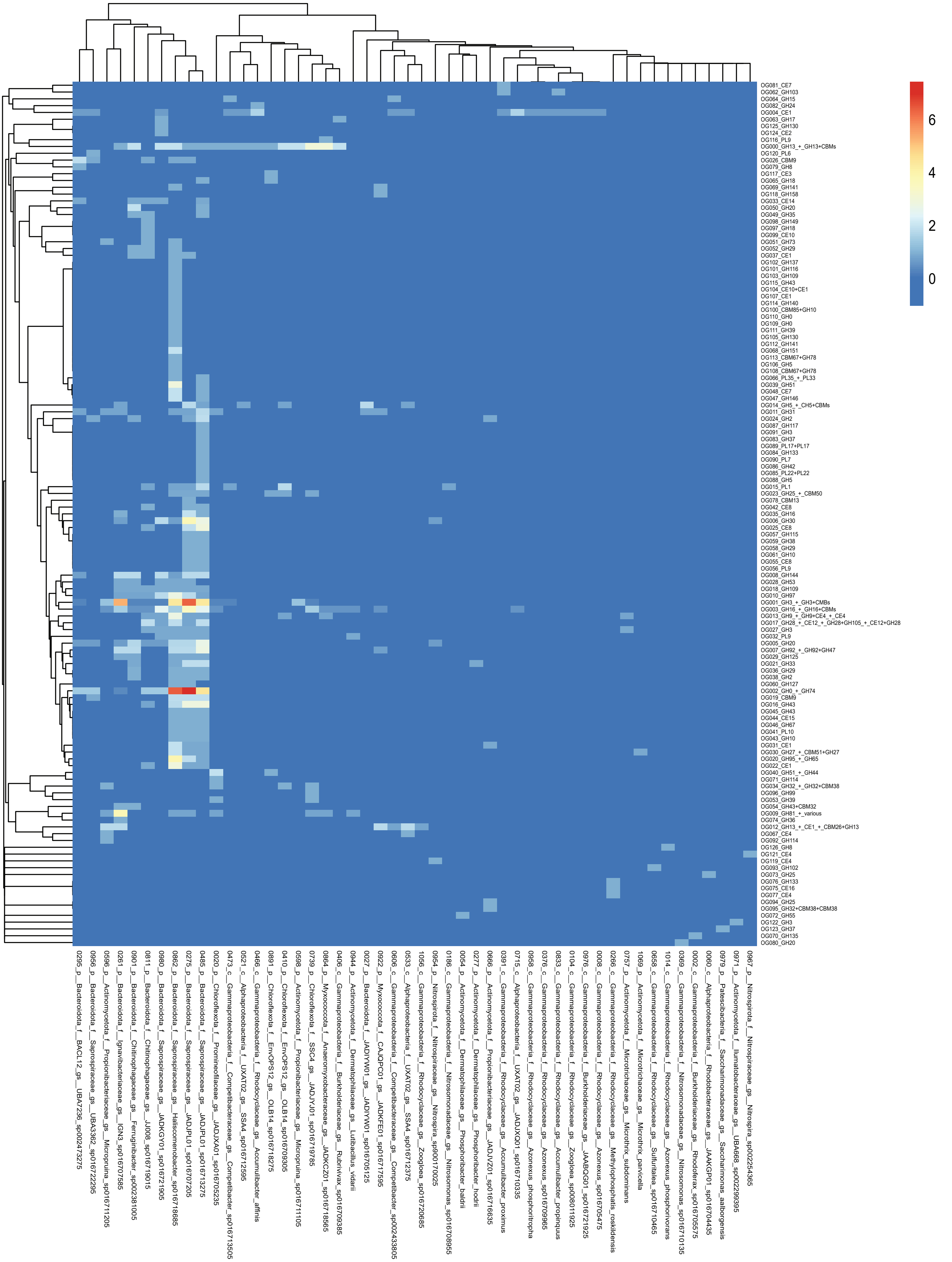

Supp. Table 7. Heatmap and cluster analysis of proteins from orthogroup analysis of predicted secreted CAZymes from “abundant and functionally relevant” MAGs (N = 63). This included proteins from all extra-cytoplasmic locations and those with Unknown locations plus signal peptides. Rows are centered; unit variance scaling is applied to rows. Both rows and columns are clustered using correlation distance and average linkage. The scale for the heatmap colours are indicated in the legend, where the scale maximum of 8 was used to enhance visualisation and differentiation of lower values <8. Note that some values were therefore >8, and raw values of OG counts are available in Supp. Table 11. The protein sequences and CAZyme annotations of the OGs are provided in Supplement Data file 3. MAG labels include the MAG number, followed by taxonomic strings of: phyla (class for Pseudomonadota), family, genus-species, denoted by p__, c__, f__, gs__, respectively.
